# Functional Olive Oil Production via Emulsions: Evaluation of Phenolic Encapsulation Efficiency, Storage Stability, and Bioavailability

**DOI:** 10.3390/nu16223909

**Published:** 2024-11-15

**Authors:** Sandra Montoro-Alonso, Carmen Duque-Soto, Ascensión Rueda-Robles, José Reina-Manuel, Rosa Quirantes-Piné, Isabel Borrás-Linares, Jesús Lozano-Sánchez

**Affiliations:** 1 Department of Food Science and Nutrition, University of Granada, Campus Universitario Cartuja s/n, 18071 Granada, Spain; sandramontoro@correo.ugr.es (S.M.-A.); jreiman@correo.ugr.es (J.R.-M.); jesusls@ugr.es (J.L.-S.); 2 Department of Analytical Chemistry, Faculty of Sciences, University of Granada, Campus Fuentenueva s/n, 18071 Granada, Spain; rquirantes@ugr.es (R.Q.-P.); iborras@ugr.es (I.B.-L.)

**Keywords:** water-in-oil (W/O) emulsion, phenolic compounds, encapsulation, in vitro digestion

## Abstract

Background/Objectives: Olive oil is valued for its health benefits, largely due to its bioactive compounds, including hydroxytyrosol (HTyr) and oleuropein (OLE), which have antioxidant, anti-inflammatory, and cardioprotective properties. However, many of these compounds are lost during the production process. This study developed a functional olive oil-derived product using water-in-oil emulsions (W/O) to incorporate commercial extracts rich in HTyr and OLE. Methods: HTyr and OLE were encapsulated in a W/O emulsion to preserve their bioactivity. The encapsulation efficiency (EE) was evaluated, and the performance of the emulsion was tested using an in vitro gastrointestinal digestion model. Bioaccessibility was measured by calculating the recovery percentage of HTyr and OLE during the digestion stages. Results: The results showed that OLE exhibited higher EE (88%) than HTyr (65%). During digestion, HTyr exhibited a gradual and controlled release, with bioaccessibility exceeding 80% in the gastric phase and a maintained stability throughout the intestinal phase. In contrast, OLE displayed high bioaccessibility in the gastric phase but experienced a notable decrease during the intestinal phase. Overall, the W/O emulsion provided superior protection and stability for both compounds, particularly for the secoiridoids, compared to the non-emulsified oil. Conclusions: The W/O emulsion improved the encapsulation and bioaccessibility of HTyr and OLE, constituting a promising method for enriching olive oil with bioactive phenolic compounds. Therefore, this method could enhance olive oil’s health benefits by increasing the availability of these bioactive compounds during digestion, offering the potential for the development of fortified foods.

## 1. Introduction

Global consumption patterns increasingly reflect a marked preference for foods enriched with functional ingredients, which confer additional health benefits beyond basic nutrition. Extra virgin olive oil (EVOO), an essential element of the Mediterranean diet, exemplifies this trend, given its well-documented potential to promote health related to its composition. EVOO is predominantly composed of triglycerides (97–99%), with monounsaturated fatty acids, particularly oleic acid, being the most abundant. Additionally, it contains significant levels of natural antioxidants, including phenolic compounds, tocopherols, and carotenoids [1,2,3]. Due to its richness in these bioactive phytochemicals, EVOO has been extensively explored for its powerful bioactive properties, establishing it as an important component of health-promoting diets.

In this sense, olive oil polyphenols have been shown to exert protection against cardiovascular disease, cancer, and neurodegenerative disorders [4,5,6,7,8], further highlighting their health-promoting potential. Specifically, hydroxytyrosol (HTyr), which is found in EVOO, and oleuropein (OLE), which is primarily found in the leaves and fruit of olive trees, have garnered significant interest due to their antioxidant properties. These phenolic compounds can act through two primary mechanisms: forming stable resonance structures through the scavenging of peroxyl radicals and peroxide chain-breaking reactions, or by preventing the copper sulphate-induced oxidation of low-density lipoprotein (LDL) [9]. In this regard, the European Food Safety Authority (EFSA) and the European Commission approved a health claim regarding the protective effects of olive oil polyphenols against blood lipid oxidation, given the presence of at least 5 mg of HTyr and its derivatives (e.g., the OLE complex and tyrosol) per 20 g of olive oil [10]. These positive effects are linked to the antioxidant protection of LDL molecules, which play a critical role in the transport and deposition of cholesterol within the body. In fact, LDL oxidation can lead to the formation of arterial plaques, which increases the risk of cardiovascular disease [11]. Thus, the antioxidant capacity of olive oil polyphenols could protect the cardiovascular system by reducing the levels of oxidized LDL due to the presence of hydroxyl groups from HTyr that can establish intramolecular hydrogen bonds with free radicals [9]. In addition to its potent antioxidant activity, HTyr has also been related to other positive beneficial effects through both in vivo and in vitro studies [12,13,14,15], such as anti-carcinogenic, neuroprotective, hypoglycemic, and anti-obesity effects, among others [16,17,18]. Concerning OLE, many of its beneficial health effects are also mainly attributable to its anti-inflammatory activity [19,20,21]. In recent years, numerous in vitro and in vivo studies have shown the important effects of OLE on inflammatory responses, which have investigated its possible role in inhibiting the production of pro-inflammatory cytokines such as IL-1β and IL6 [19]. These anti-inflammatory effects have been attributed to its ability to inhibit the activation of key signalling pathways involved in the inflammatory response, including the NF-κB and MAPK pathways [22,23]. 

As a result of their bioactive potential, a growing interest in the incorporation of these compounds into functional foods has led to a focus on strategies to enhance their bioavailability and consequently their bioactivity. Moreover, these phenolic compounds are often present at low concentrations in EVOO while being affected by various factors, such as oxygen, light, and temperature, which result in a significant degradation during the oil extraction process, limiting their shelf life and bioavailability [24]. Consequently, encapsulation techniques offer a strategic approach to mitigate their loss by stabilizing the phenolic compounds and protecting them from degradation. In this context, emulsion-based delivery systems have become one of the leading encapsulation methods, offering numerous benefits for the incorporation of natural antioxidants into food products [25,26]. These benefits include improved chemical stability and the potential to boost bioavailability, fortification, or both.

In particular, water-in-oil (W/O) emulsions are widely used across the food, pharmaceutical, and cosmetic industries to efficiently encapsulate and deliver bioactive compounds [27,28,29,30]. A W/O emulsion consists of oil as a continuous phase with dispersed water droplets, which are stabilized by lipophilic emulsifiers to maintain a uniform distribution. This colloidal system enables the efficient encapsulation of both lipophilic and hydrophilic compounds in various food formulations, while also protecting bioactive compounds from degradation in the gastrointestinal tract [31]. This approach could enable the development of functional edible oils enriched with well-preserved phenolic compounds. These formulations could ensure a more consistent dietary intake, enhance their bioactive potential, and allow for the controlled release of these compounds, providing sustained and prolonged delivery.

Thus, the present study focused on the development of a functional olive oil product using W/O emulsions to incorporate HTyr- and OLE-rich commercial extracts. Furthermore, the encapsulation efficiency (EE), stability over time, and bioaccessibility of the main phenolic compounds in the W/O emulsion were also assessed. In this sense, understanding the factors impacting the bioaccessibility of polyphenol emulsions can provide valuable insights for developing functional foods with enhanced health benefits. In addition, the encapsulation and its evolution during the storage of the extracts within the oil matrix were evaluated.

## 2. Materials and Methods

### 2.1. Samples

EVOO samples consisted of multivarietal blends of 33% of Arbequina, Picual, and Hojiblanca olives harvested during the crop season of 2023/2024. These samples were supplied by the Torres Morente S.A.U company located in Escúzar (Granada, Spain). Olives were processed in an industrial plant equipped with a hammer crusher, horizontal blender, one horizontal and one vertical centrifuge (two-phase system), and a conical decanter. Samples were stored in amber bottles without headspace in the dark at room temperature until analysis.

### 2.2. Chemicals and Reagents

Commercial olive leaf extract with HTyr (20% *w*/*w*) and OLE (35% *w*/*w*) were kindly provided by Deretil S.L. (Cuevas del Almanzora, Spain). Polyglycerol polyricinoleate (PGPR) was purchased from Savannah (Barcelona, Spain). LC-MS-grade methanol and HPLC-grade n-hexane were purchased from Honeywell (Charlotte, NC, USA), whereas ethanol, ethyl acetate, and LC-MS-grade acetic and formic acids were obtained from Scharlab (Barcelona, Spain) and Sigma-Aldrich (Steinheim, Germany), respectively. Milli-Q water was purified using a Milli-Q system (Millipore, Bedford, MA, USA). Commercially available pure standards were acquired for both qualitative and quantitative purposes. Trans-p-coumaric acid, HTyr, tyrosol, and OLE were purchased from Sigma-Aldrich (St. Louis, MO, USA), and (+)-pinoresinol was acquired from PhytoLab (Vestenbergsgreuth, Germany). Apigenin and luteolin were purchased from LGC (Teddington, Middlesex, UK), and luteolin 7-O-glucoside, loganin, and verbascoside were purchased from Extrasynthese (Lyon, France).

For in vitro digestion, the enzymes pepsin 3412 U/mg and pancreatin 4 × USP and bovine bile salts (Sigma B-3883) were purchased from Sigma-Aldrich (Saint Louis, MO, USA), whereas rabbit gastric extract was acquired from Lipolytech (Marseille, France). Chemicals for the preparation of simulated digestive fluids, hydrochloric acid (HCl, 37%), calcium chloride (CaCl_2_(H_2_O)_2_), sodium hydroxide (NaOH), sodium chloride (NaCl), sodium bicarbonate (NaHCO_3_), potassium dihydrogen phosphate (KH_2_PO_4_), potassium chloride (KCl), magnesium chloride hexahydrate (MgCl_2_(H_2_O)_6_), and ammonium carbonate ([(NH_4_)_2_CO_3_]), were purchased from Sigma-Aldrich (Steinheim, Germany).

### 2.3. Preparation of the W/O Emulsion

The W/O emulsions were prepared with olive leaf extracts according to the method described by Fregapane et al. [32] with some modifications. The W/O emulsion consisted of 97.5% (*w*/*w*) EVOO, 2% (*w*/*w*) water, and 0.5% (*w*/*w*) PGPR as a surfactant. Before emulsification, PGPR was mixed with EVOO to form the oil phase (O phase), whereas the olive leaf extracts were dissolved in water to form the aqueous phase (W phase), resulting in a concentration of 25 mg of HTyr and OLE per 100 g of emulsion. The dispersed phase was added dropwise to the continuous phase and the mixture was stirred for 5 min at 300 rpm. The emulsification was carried out using a high-intensity ultrasonic homogenizer (UP400St, Hielscher Ultrasonics, Germany) and a 14 mm diameter sonotrode (s24d14D, Hielscher Ultrasonics, Teltow, Germany). To produce fine emulsions, the amplitude was fixed at 46% and the temperature was set below 40 °C. After homogenization, the emulsions were distributed into individual glass containers and stored at room temperature for the later assessment of both the EE and phenolic stability over time.

#### Stability Under Storage

In order to evaluate the stability of the obtained formulation, samples were taken at 0, 4, 8, 12, 16, and 19 days (T0, T4, T8, T12, T16, and T19, respectively) of storage at room temperature, for assessing the phenolic content and EE of both EVOO and the W/O emulsion. Thus, samples were extracted as described in Section 2.6 and characterized using HPLC-QTOF-HRMS, as described in Section 2.7.

### 2.4. Simulated In Vitro Gastrointestinal Digestion

EVOO, the emulsion, and olive leaf extract were subjected to static in vitro gastrointestinal digestion following the recently updated harmonized INFOGEST method [33]. The oral phase was performed by mixing 5 g of the samples with 5 mL (1:1, *w*/*v*) of simulated salivary fluid (SSF) in a 50 mL conical centrifuge tube, protected from light. The mixture was incubated at 37 °C and pH 7.0, shaking for 5 min at 55 rpm (Onilab MX-RD-Pro, Labbox, Barcelona, Spain). For the gastric digestion phase, 10 mL of simulated gastric fluid (SGF) containing 2000 U/mL of pepsin and 60 U/mL of gastric lipase were added to the mixture. The pH was adjusted to 3.0 by adding 1 M HCl, and Milli-Q water was added to achieve a final volume of 20 mL. The mixture was homogenized and incubated for 2 h at 37 °C with constant agitation at 55 rpm. Finally, simulated intestinal fluid (SIF, 20 mL) containing bile salts (10 mM) and pancreatin (100 U/mL) was added to the previous mixture and stirred for 2 h at pH 7 and 37 °C. Milli-Q water was added to obtain a final volume of 40 mL.

Samples were collected at 30, 60, and 120 min (gastric phase) and 180, 210, and 240 min (intestinal phase), respectively. At the end of each digestion phase, the obtained samples were centrifuged for 10 min at 8874× *g* to separate the water phase from the oily phase and stored at −20 °C until further analysis. The bioaccessible fraction, which contained phenolic compounds, was obtained at the end of the intestinal digestion.

The cumulative presence of the bioaccessible fraction throughout the digestion process was calculated as previously described ([34], using Equation (1)). This calculation expresses the bioaccessible fraction as a percentage of the initial composition, based on the quantity measured in the chemical extracts of the analyzed samples:

(1) $$Recovery\left(\%\right)=\frac{PC\;content\;in\;DS\;(mg)}{Initial\;PC\;content\;(mg)}\times 100$$

where PC represents phenolic compounds, DS refers to the digested samples for each phase, and the initial PC content corresponds to the phenolic content present in the EVOO, emulsion, and extract samples for each case.

### 2.5. Extraction of Phenolic Compounds from EVOO, Emulsions, and Digested Samples

The extraction of polyphenols from EVOO and the emulsions was carried out as described by López-Salas et al. [35]. Briefly, 5 g of EVOO or the emulsion was dissolved in 10 mL of n-hexane and homogenized. After adding 10 mL of methanol–water (60:40, *v*/*v*), the mixture was vortexed and centrifuged at 1150× *g* (Sorvall ST 16, Thermo Scientific, Barcelona, Spain) for 10 min at 4 °C to separate the fractions. This extraction was repeated twice. The combined aqueous extracts were evaporated to dryness in the rotary evaporator at a temperature below 40 °C, and the residue was dissolved in 0.5 mL methanol–water (50:50, *v*/*v*), diluted to 1:5 (*v*/*v*), and finally filtered through a 0.45 µm filter before HPLC-MS analysis.

The extraction of non-encapsulated phenolic compounds was achieved according to previous studies with some modifications [36]. Briefly, 1 mL of the W/O emulsion was mixed with 5 mL of water and gently stirred. The mixture was then centrifuged at 986× *g* for 10 min at 25 °C, and the supernatant was evaporated to dryness in a rotary evaporator at temperatures below 40 °C. The obtained residue was dissolved in 0.5 mL of methanol–water (50:50, *v*/*v*), diluted to 1:5 (*v*/*v*), and finally filtered through a 0.45 µm filter before HPLC-MS analysis. Given its natural presence in the continuous phase of the emulsion, the EE of HTyr was calculated using the following equation, Equation (2):

(2) $$EE\;HTyr\;(\%)=\frac{\left({HTyr}_{TE}-{HTyr}_{TO})-({HTyr}_{NEE}-{HTyr}_{NEO}\right)}{{HTyr}_{TE}-{HTyr}_{TO}}\times 100\;$$

where *HTyr_TE_* is the total *HTyr* concentration in the emulsion, *HTyr_TO_* is the total *HTyr* concentration in the starting oil, *HTyr_NEE_* is the non-encapsulated concentration of HTyr in the emulsion, and *HTyr_NEO_* is the concentration of *HTyr* obtained from the oil following the extraction method to obtain the non-encapsulated fraction.

On the other hand, for OLE, since it is not present in EVOO, Equation (3) was used to determine its EE as follows:

(3) $$EE\;OLE\;\left(\%\right)=\frac{{OLE}_{TE}-{OLE}_{NEE}}{{OLE}_{TE}}\times 100$$

where *OLE_TE_* is the total *OLE* concentration in the emulsion and *OLE_NEE_* is the non-encapsulated *OLE* concentration.

The extraction of phenolic compounds from digested samples (EVOO, the emulsion, and olive leaf extracts) was performed following the method reported by Juániz et al. [37] with some modifications. Digested samples collected at different times were extracted by mixing 4 mL of the gastric water phase and 8 mL of the intestinal water phase with 4 mL and 8 mL of ethyl acetate, respectively. The mixture was then vortexed and centrifuged at 15,776× *g* for 10 min at 4 °C to separate the fractions. This extraction was repeated twice. The combined extracts were evaporated to dryness in a rotary evaporator at a temperature below 40 °C, and the residue was dissolved in 0.5 mL methanol–water (50:50, *v*/*v*) and diluted to 1:5 (*v*/*v*) for the emulsions and olive leaf extracts before being filtered and analyzed through HPLC-MS analysis.

### 2.6. Bioactive Compound Characterization Using HPLC-QTOF-HRMS

The analysis of samples was conducted using a previously reported method [38]. Qualitative and quantitative analyses of the phenolic compounds were carried out using an HPLC system coupled with a quadrupole time-of-flight high-resolution mass spectrometer QTOF-HRMS (Bruker Daltonik, Bremen, Germany). The QTOF mass analyzer was equipped with an ESI interface operating in negative ion mode in a mass range of 50–1000 *m*/*z*. The analytical column used for separation was a Zorbax Eclipse Plus C18, 150 mm × 4.6 mm internal diameter, 1.8 µm (Agilent Technologies, Palo Alto, CA, USA). The mobile phases consisted of water plus 0.25% acetic acid (Solvent A) and methanol (Solvent B) eluted according to the following multistep gradient: 0 min, 5% Solvent B; 7 min, 35% Solvent B; 13 min, 45% Solvent B; 18.5 min, 50% Solvent B; 22 min, 60% Solvent B; 29 min, 95% Solvent B; 36 min, 5% Solvent B. The flow rate was 0.5 mL/min, the temperature was maintained at 25 °C, and the injection volume in the HPLC system was 5 μL. The external calibration of the mass spectrometer was performed using sodium formate solution as the calibrant, which was prepared as follows: 5 mM sodium hydroxide and 0.2% formic acid in water–isopropanol (1:1, *v*/*v*). The calibration solution was injected at the beginning of each run, and all spectra were calibrated before compound identification.

To quantify the analytes identified in the samples, duplicate injections of each extraction replicate were conducted for each sample type. This approach was implemented to ensure the reproducibility of both the extraction process and the analytical measurements. Quantification was performed using calibration curves prepared using the standard compounds. Stock solutions of each of these standards (apigenin, luteolin, luteolin 7-O-glucoside, loganin, verbascoside, OLE, HTyr, tyrosol, pinoresinol, and coumaric acid) were prepared in methanol at a concentration of 1000 ppm and stored at −20 °C. Then, solutions of different concentrations of these standards were prepared in the same solvent at concentrations of 0.1, 0.5, 1, 5, 10, 25, 50, 75, 100, 120, and 150 mg/L. The concentrations of the identified phenolic compounds were determined by interpolating the peak areas from replicate analyses of each sample into appropriate calibration curves, presented in the Supplementary Materials (Table S1). The other phenolic compounds, which had no commercial standards, were tentatively quantified with other compounds having similar or related structures. Thus, the phenolic content was expressed as the mean concentration ± standard deviation for each sample expressed as mg compound/kg sample (Table S2). After the analysis of the samples, the data were processed using DataAnalysis 6.1 and TASQ^®^ software 2023 0.5.857 (Bruker Daltoniks, Bremen, Germany).

### 2.7. Statistical Analysis

The experiments were performed in triplicate and comparisons were made using the SPSS statistical software (SPSS version 28; SPSS Inc., Chicago, IL, USA). Analysis of variance (ANOVA) and Tukey’s post hoc tests with α at 0.05 were applied to determine statistical differences among conditions and digestive phases at a 95% confidence level.

## 3. Results and Discussion

### 3.1. Qualitative and Quantitative Characterization of Phenolic Compounds in EVOO, Olive Leaf Extract, and W/O Emulsions

**Figure 1:**
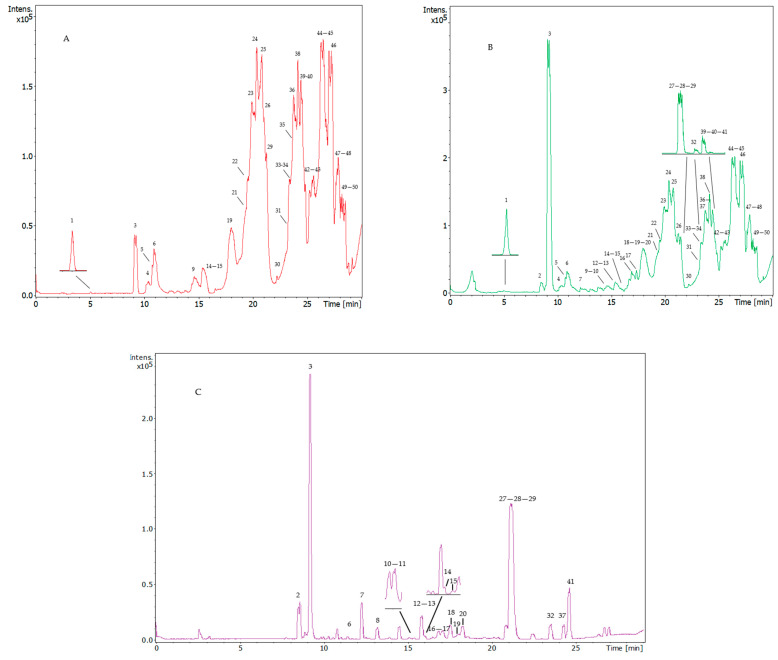

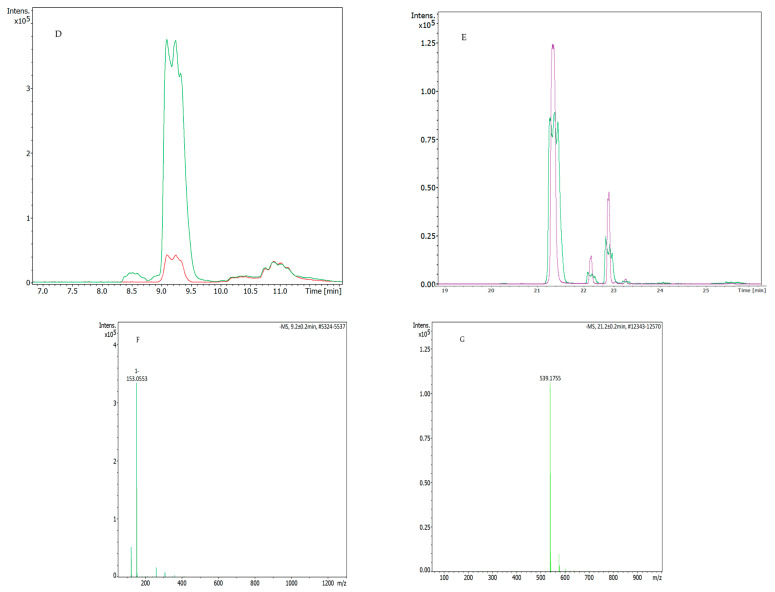
Representative base peak chromatogram for EVOO (**A**), W/O emulsion (**B**), and olive leaf extract (**C**) extracts, EIC of HTyr in EVOO (red) and W/O (green) samples (**D**) and OLE in W/O emulsion (green) and olive leaf extract (purple) (**E**), and mass spectrum of HTyr (**F**) and OLE (**G**).

This study characterized and quantified the phenolic compounds in EVOO, olive leaf extract, and emulsions enriched with HTyr and OLE. As shown in Table 1, a total of 50 phenolic compounds and derivatives were identified in the oil, the emulsion, and the incorporated extract. Among these, five compounds were phenolic alcohols, thirty belong to the secoiridoids family, three were lignans, four correspond to flavonoids, and eight substances were oleosides and elenolic acid derivates. These compounds were characterized using the chemical information provided by the HPLC-QTOF-HRMS instrument (Figure 1). Some of the detected compounds were tentatively identified by comparing their retention times and mass spectra with commercially available standards. All other compounds lacking available commercial standards were identified by interpreting their mass spectra and molecular formulas using DataAnalysis 6.1 software (Bruker Daltoniks, Bremen, Germany). This information was further complemented by data previously published in databases and the literature related to olive oil and leaf composition.

Quantitation information for each sample of individually identified compounds is reported in Table 2. Concerning the phenolic alcohols, HTyr and tyrosol were the predominant simple phenols identified in the EVOO samples. The average concentrations of HTyr and tyrosol were 14 ± 1 mg/kg and 7.7 ± 0.5 mg/kg, respectively. These findings are consistent with the reported literature on the phenolic content of olive oils [39]. Owen et al. [40] reported mean concentrations of 14 mg/kg for HTyr and 28 mg/kg for tyrosol. While the HTyr concentrations are consistent with these findings, the tyrosol concentration of the analyzed samples is notably lower. However, Bayram et al. [41] reported tyrosol concentrations ranging from 3.6 to 38.4 mg/kg, suggesting that the present tyrosol concentrations fall within the broader range observed by other researchers. Similarly, De la Torre-Carbot et al. [42] reported a broader range of concentrations, from 7 to 64 mg/kg for HTyr and from 3 to 24 mg/kg for tyrosol, which are indicative of the considerable variability across different olive oils. This variability in the HTyr and tyrosol content can be largely attributed to differences in olive varieties, agronomic conditions, olive ripeness, oil extraction and processing techniques, and post-harvest practices [43,44,45,46].

Concerning emulsions, the HTyr concentration was 221 ± 10 mg/kg, which indicates a significant enrichment of the emulsified oil compared to its base matrix (14 ± 2 mg/kg). Despite that, a slightly significant increase was also found in the concentration of oxidized HTyr. The phenolic alcohol composition also reported the HTyr glycosylated form since this compound was characterized in the extract used for emulsion formulation.

In addition to HTyr and its derivatives, a significant increase in the tyrosol concentration was observed in the emulsion compared with the olive oil samples (9.3 ± 0.1 mg/kg vs. 7.7 ± 0.5 mg/kg, respectively). This increase could have a dual origin: on the one hand, the tyrosol present in the incorporated enriched extract (Table 2), and on the other hand, the tyrosol produced due to the hydrolysis of ligstroside aglycone derivatives as a consequence of the emulsification process. Indeed, a decrease in oleocanthal (decarboxymethyl ligstroside aglycone) concentrations was also recorded in the emulsion when compared to the content in EVOO.

Regarding the secoiridoid group, the presence of oleuropein aglycone (OA) isomers is notable in EVOO samples (Table 1). OLE is mainly found in the leaves and fruits of olive trees, and also in EVOO, in its glycosidic form because of the action of the β-glucosidase released during the crushing of olive drupes. This enzyme favours the breakdown of the glycosidic bond, giving rise to the generation of aglycones [47]. Moreover, other compounds identified in the EVOO include oleacein (72 ± 8 mg/kg), a compound generated during the oil extraction process and the ripening of olive fruit [48,49], and ligstroside aglycone isomers (LA), whose concentrations were slightly higher than those obtained by other authors [50]. These differences may be due to the variations in three production factors: the time of malaxation applied during the process of oil extraction [44], the temperature of trituration, and the state of maturation of the olive fruit [51].

Compared to EVOO, a significant decrease in the OA content in the emulsion was observed, which could be attributed to the emulsification process. However, it is important to remark that a supplementation of OLE was developed through the formulation. The OLE content of the emulsion obtained was 89 ± 0.5 mg/kg, and two isomeric forms of this compound were also identified in both the olive leaf extract and the emulsion. Overall, OLE is not naturally present in the oil and only some authors have detected it in this matrix in very low concentrations, ranging from 0.06 mg/kg to 1.66 mg/kg, values below those obtained for our W/O emulsion samples [52].

On the other hand, lignan and flavonoid concentrations in the analyzed EVOO and emulsions were similar to those determined in other EVOO samples. On the contrary, the pinoresinol concentrations determined for the emulsion were significantly lower compared to those of the starting oil, (7.0 ± 0.3 mg/kg vs. 6.5 ± 0.1 mg/kg), whereas the luteolin concentrations were similar in both samples.

For the group of oleosides and derivatives of elenolic acid, there was a significant increase in the concentration of the elenolic acid isomer 2 in the emulsion in respect to EVOO. Although this compound is present in the olive leaf extract used for the enriched formulation, part of this increase may also be derived from the hydrolysis of the secoiridoids generated during the emulsification process. In the emulsion, compounds were also found belonging to the composition found in the incorporated extract, such as diol geminal OLE aglycone isomers or the glycosylated form of elenolic acid (Table 2).

### 3.2. Monitoring the Encapsulation Efficiency and Phenolic Profile During Storage

**Figure 2:**
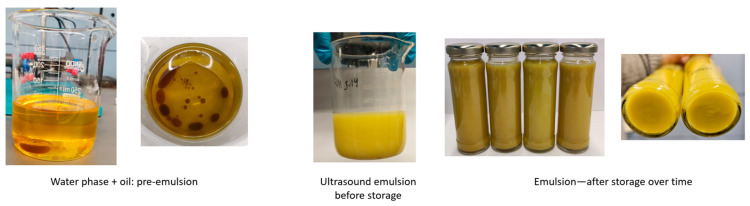
Emulsification process and stability over time, showing the initial oil phase, mixing stages, fully emulsified sample, storage, and final emulsion after storage with no phase separation.

Being an outstanding source of phenolic compounds with potential health benefits, the incorporation of the evaluated extract for the obtention of a functional olive oil product was performed (Figure 2). In this sense, the aim of this inclusion was to increase the bioactive content for ensuring a presence superior to 5 mg of HTyr and its derivatives and, thus, surpassing the requirements for the previously stated EFSA claim. Hence, to evaluate the potential of the obtained formulation, the EE and phenolic content evolution of both EVOO and the W/O emulsion were assessed. In this sense, the main phenolic compounds identified in both matrices were quantified at different times, presented in the Supplementary Materials (Table S2).

Regarding the EE of HTyr and OLE, both compounds were successfully incorporated, with OLE exhibiting a higher EE compared to HTyr (88 ± 6% and 65 ± 6%, respectively). The lower efficiency observed for HTyr, a water-soluble compound with a partition coefficient of log P_o/w_ = 1.1 (log P_o/w_ refers specifically to the partition coefficient between octanol (o) and water (w)) [53,54], could be related to a partial migration into the oil phase. Since the low value of log P_o/w_ indicates that HTyr is slightly more soluble in octanol (lipophilic) than in water (hydrophilic), its migration into the lipophilic fraction of the emulsion could be explained. Furthermore, HTyr may interact with molecules in the surfactant monolayer, hindering its encapsulation within the W/O formulation [53,55]. Nevertheless, the results for HTyr incorporation are consistent with the previous literature, where this compound was successfully incorporated into various W/O emulsions, although specific EE values were not reported [53,56]. In this regard, the encapsulation of HTyr derivatives may improve EE. For example, Caceres et al. [57] reported an EE of 87% for alkyl esters of HTyr in walnut oil, although the inclusion of a spray-drying step may have contributed to this increased efficiency.

With respect to other emulsion-based approaches, the presented data appear to be comparable to those reported for double emulsions. In Flaiz et al. [54], the encapsulation of HTyr in multiple emulsions resulted in a lower EE (55%), which may be attributed to a reduction in the presence of this antioxidant within a more compartmentalized system, potentially due to an increase in surface area content.

As for OLE, the high EE observed exhibits an adequate incorporation of this compound into the encapsulated formulation. Indeed, these results are comparable to those presented in the literature for different emulsion-encapsulating approaches to OLE, with values up to 91% [58,59].

Overall, EE values can be highly variable depending on several factors, including the technology, encapsulating agents, and the nature of the encapsulated compound, and may not be ideal for the development of different food products. Thus, although multiple encapsulation technologies could be used for the target phenolic compounds, the selected methodology allows for a phenolic-enriched product formulation while considering the nature of the oil matrix through a green process.

**Figure 3:**
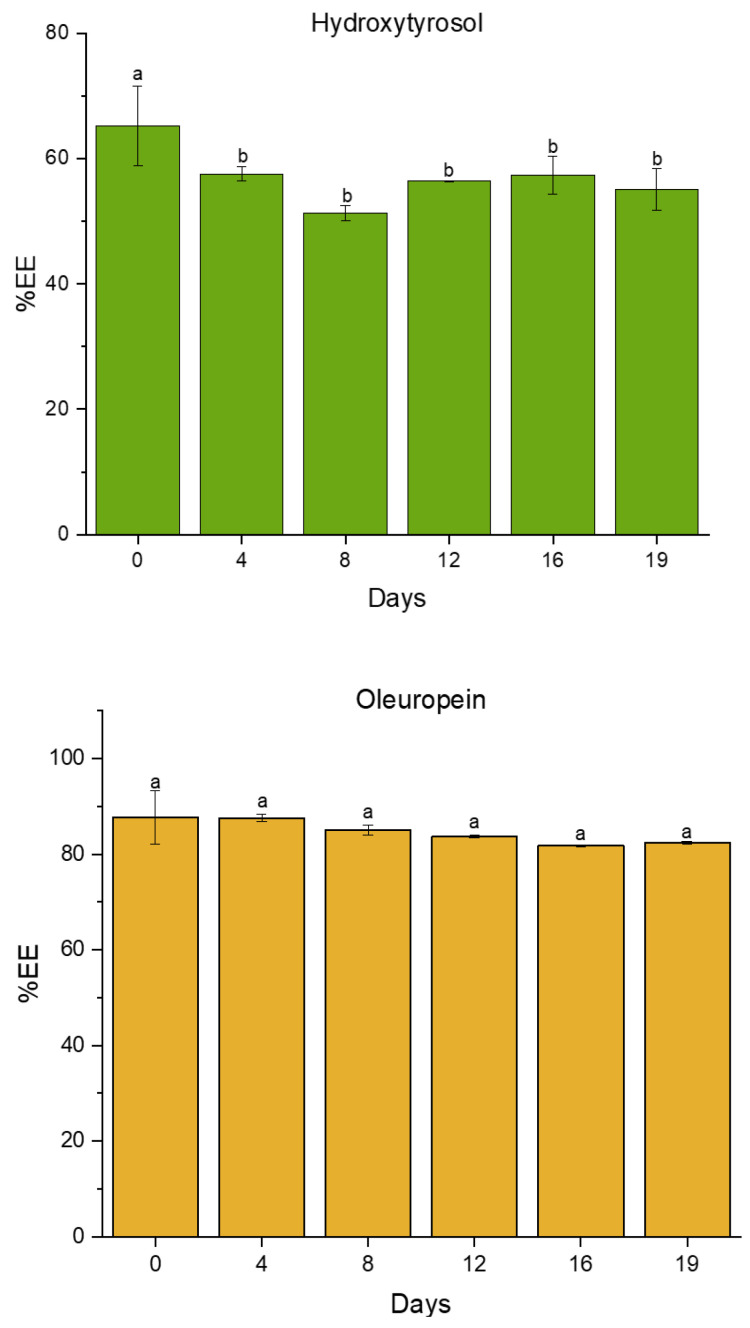
Encapsulation efficiency (%EE), expressed as a percentage, for hydroxytyrosol (green) and oleuropein (yellow) over a 19-day period (days). EE was calculated as described in the Section 2. Values with different superscript letters within each diagram are significantly different at *p* < 0.05.

Additionally, the evolution of the EE for both compounds was evaluated over 19 days of storage, as depicted in Figure 3. For the phenolic alcohol, the EE values were slightly reduced after the formation of the emulsion (day 0), showing a significant reduction that is maintained throughout the storage. This decrease could be attributed to various factors, such as a mild degradative effect associated with the emulsification process. Additionally, as previously mentioned, as a result of its partition coefficient, this compound may partially be transferred into the lipid continuous phase, with the release of a reduced percentage of the compound that was previously encapsulated, or be more externally located at the water–oil interface [54]. Indeed, the stability of the EE for this compound after 4 days of storage seems to indicate the absence of a significant degradative effect of the encapsulation process. In this regard, the evolution of the EE for HTyr is promising compared to the previous literature for other encapsulation techniques, where a partial release of the compound is often observed during storage. Thus, in Yuan et al. [60], EE values for HTyr encapsulated in liposomes were reduced from 45.08% to 41.5% after 15 days.

On the contrary, the EE of OLE remained stable throughout the storage period, showing no significant differences, indicating optimal encapsulation and retention with high efficiency values. These findings differ from the previous literature reports. For instance, Robert et al. [61] observed a slight decline in the OLE content after 14 days of storage, from 92.0 ± 3.1% to 82.3 ± 2.6%, though the values remained relatively high. In contrast, double W/O/W emulsions loaded with OLE exhibited approximately a 40% release of the total content after 28 days of storage.

**Figure 4:**
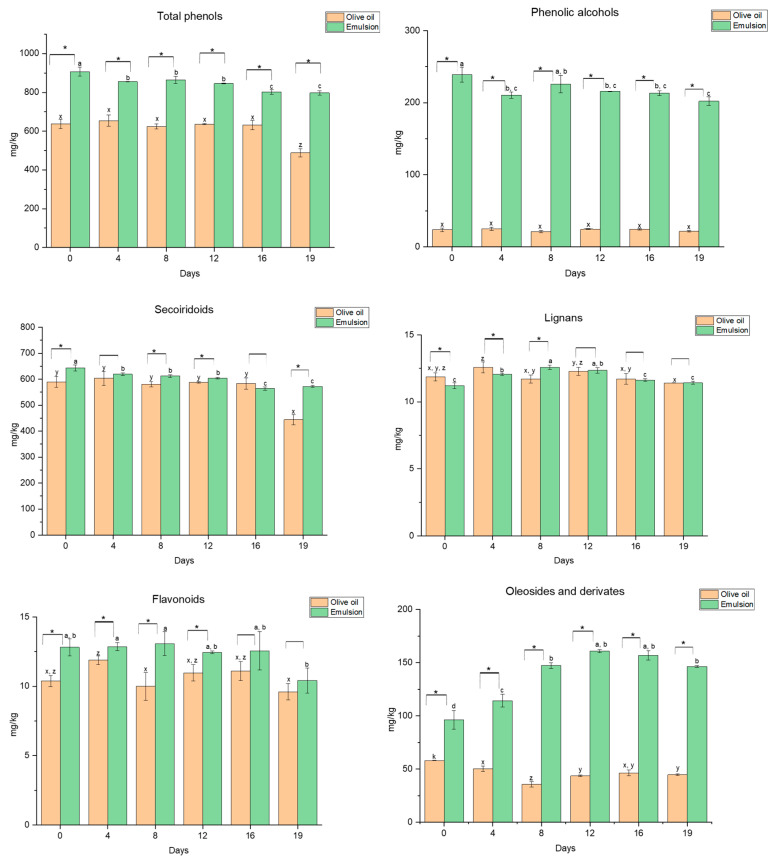
Total concentration and evolution over time (days) of the total phenols, phenolic alcohols, secoiridoids, lignans, and oleosides and elenolic acid derivates in the starting EVOO and the emulsion expressed as a mg/kg compound. ANOVA statistical analysis is represented using letters to compare the results of the time in the samples over the days of storage (*p* < 0.05). Different letters indicate significant differences. Asterisks are used to indicate a statistically significant difference between the two matrices on each day of storage.

Additionally, the phenolic profile of both the EVOO and the W/O emulsion was monitored over the storage period (Figure 4). Thus, the incorporation of the phenolic extract resulted in an increase in the total phenolic content, which remained consistent throughout the evaluated period. Although a slight reduction was observed at the end of storage, the significant differences between the enriched EVOO and the control were maintained, indicating successful enrichment.

In concordance with the observed trend for the total phenolic content, the presence of phenolic alcohols is significantly increased in the emulsion, resulting from the inclusion of the phenolic extract. In respect to their behaviour during storage, differences can be found between the evaluated matrices. Firstly, EVOO appears to be a stable matrix for its naturally present phenolic compounds with no significant differences during storage. However, in the emulsion, a fluctuation in the phenolic alcohol content can be observed during the initial phase of storage and stabilizes by day 12, with the concentration remaining above 200 mg/kg throughout the storage period.

As the most representative compound of this family, the HTyr content in the EVOO seems to remain stable over the period studied. In the emulsion, this is achieved after a slight decrease at the beginning of storage (data in the Supplementary Materials). As described above, these results could be related to phenomena occurring during the emulsification process. In this respect, a reduction in the content of encapsulated HTyr has already been reported in both single and double emulsions over a period of 22 days, with a decrease of 8.6% and 26.9%, respectively [54].

Similar behaviour has been observed for other related compounds, such as HTyr acetate, while tyrosol remains fairly stable. Nevertheless, fortification resulted in a significant increase in the HTyr content, with its content being twelve times higher than that observed for EVOO during storage.

In addition, the behaviour of secoiridoids in both EVOO and the emulsions varied during storage. In the oil, an initial stability was observed, followed by a significant decrease towards the end of the evaluation period, primarily attributed to the degradation of oleuropein aglycone, ligstroside aglycone, and D-oleuropein aglycone (DOA). Secoiridoid levels in the emulsions exhibited a consistent stable trend over time, with only minor variations observed over the storage period (Figure 4). Nevertheless, the overall secoiridoid content in the emulsions remained comparable to that of the oil, with no significant differences observed throughout most of the storage days. Concerning secoiridoid derivatives, including hydroxylated DOA, hydrated DOA, and methylated DOA, these also showed slight reductions, although the decreases were minimal. In spite of this, the secoiridoid content remained significantly higher in the emulsion for most of the period under consideration.

These findings indicate that the emulsion matrix provides superior protection for secoiridoids compared to EVOO, likely due to its ability to mitigate the oxidation or degradation of these compounds. The stability observed in the emulsion up to day 12, followed by a gradual decline, suggests its effectiveness in preserving the bioactive properties of secoiridoids during storage. In contrast, the significant reduction in EVOO after 16 days highlights its susceptibility to degradation during prolonged storage.

Thus, the OLE content demonstrated a stabilized general trend in the emulsion, consistent with the previously reported EE. Moreover, both isomers 2 and 3 showed slight decreases but remained stable from the eighth day onwards. This stability pattern is in alignment with findings from González-Ortega et al. [62], where OLE encapsulated in liposomes displayed an initial decline, followed by stabilization over a 500 h (20.8 days) storage period. On the other hand, the slight reduction in secoiridoids in the emulsion may be related to a decrease in other constituents, such as OLE aglycone and ligstroside aglycone. However, in all cases, these compounds were stable after the 16th day, suggesting a stabilization of these compounds and a non-lasting effect of the encapsulation process.

Our results are in agreement with those observed in Jolayemi et al. [63], where the incorporation of a free olive phenolic extract into a corn oil-based salad dressing through a W/O emulsion process resulted in an average decrease of 8% of the total phenolic content at the beginning of storage, which remained constant during the rest of the evaluated period. This decrease was also observed in the W/O nanoemulsions of an açai berry extract, with a retention of >70% of the initial phenolic content after 30 days of storage at 4 °C [64]. Indeed, in our study, the retention of these compounds is in line with these data, with only a reduction of 15–20% when stored at room temperature (25 °C).

As previously mentioned, the slight initial decrease in some of the evaluated compounds could be associated with the emulsification process. In this sense, the cavitation phenomenon inherent to this methodology is characterized by the formation of localized areas of increased temperature and pressure, which may potentially enhance the physical and chemical reactivity of phytochemicals. In particular, this effect can lead to the generation of radicals, such as •OH and •H, which may initiate the degradation of bioactive compounds present in the system related to an antioxidant response [65]. Thus, a reduction in these compounds may be related to an antioxidant response [66]. However, due to the transient nature of this phenomenon, it can be concluded that it is unlikely to have any effect on these compounds beyond the first few days after emulsion preparation. Thus, the applied methodology appears to be suitable for the incorporation of bioactive compounds into the selected lipophilic matrix through a green process.

The evolution over time of the fortification of other phenolic families not related to the main compounds on which this study focused was also evaluated. The trend of lignans in both EVOO and the emulsion shows significant differences during the early storage stages. At T0 and T4, the lignan content is significantly higher in the oil than in the emulsion (*p* < 0.05). However, by T12, no significant differences are observed between the two matrices. Over time, lignan levels in both the oil and emulsion decrease, stabilizing after T8. This behaviour suggests that the oil offers some protection against lignan degradation early on, while both matrices reach a similar lignan content in the later storage stages. Furthermore, flavonoids also presented high stability during storage with a significantly higher content in the emulsion as a result of the content of luteolin 7-glucoside of the added extract. Nevertheless, a reduction can be observed in both matrices (emulsion and EVOO) at the end of storage.

With regard to non-phenolic compounds, oleoside and elenolic acid derivatives increased in the emulsion, although their content varied. Their presence is related to the degradation of other phenols, mainly secoiridoids. In EVOO, oleoside levels remained consistently lower and relatively stable after 8 days, indicating less protection and potential early degradation compared to the emulsion.

Overall, fortification through emulsification has led to a positive increase in olive phenolic compounds. Indeed, the phenolic alcohol content is 10 times higher than in the non-emulsified oil. Although the evolution of these compounds in the emulsion may lead to a similar trend to that in EVOO, significant differences are maintained between both matrices for the HTyr and derivates and oleoside/elenolic acid derivatives, which remain 14 times and 1.7 times higher in the fortified emulsion. This fact, together with the subsequent stability of the target compounds at the end of storage, makes emulsification an under-explored but potential technique for obtaining fortified formulations of vegetable oils with an increased content of beneficial phenolic compounds.

### 3.3. In Vitro Digestion

To evaluate the potential of the obtained formulation in terms of HTyr and OLE bioaccessibility, both the source materials (EVOO and olive extract) and the W/O emulsion were subjected to an in vitro gastrointestinal digestion process simulating the oral, gastric, and intestinal phases. Thus, the stability and bioaccessibility of HTyr and OLE under gastrointestinal conditions were assessed to monitor their metabolic transformation and liberation from the considered matrices.

**Figure 5:**
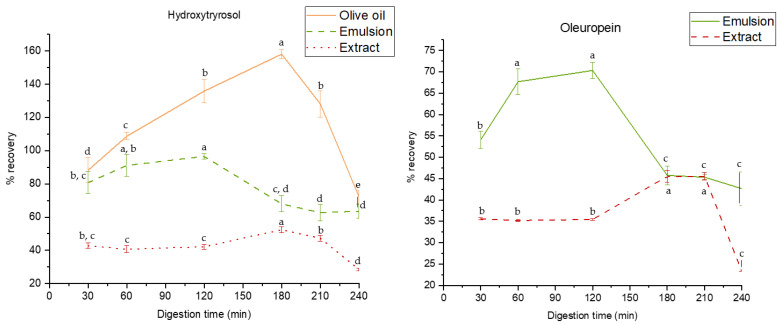
Evolution of hydroxytyrosol and oleuropein in extra virgin olive oil, emulsion, and extract under gastric (30, 60, and 120 min) and intestinal (150, 180, and 240 min) phases of in vitro gastrointestinal digestion. ANOVA statistical analysis is represented using letters to compare the results of the time in the samples throughout the digestion process.

The accumulative presence of the target compounds in the bioaccessible fraction throughout the digestion process was calculated as a percentage of their initial composition [34]. The evolution of the recovery percentage for these phenolic compounds, providing insights into the fraction potentially available for absorption, is presented in Figure 5.

In this sense, HTyr exhibited a high recovery percentage during the initial stages of digestion (exceeding 100%) in the oil, reaching a peak value of 158% at 180 min of intestinal digestion. This pronounced recovery could be attributed to its sensitivity to phase transitions, particularly at the gastric–intestinal interface. This sharp increase may result from chemical transformations induced by changes in the environmental conditions, such as the pH and enzymatic activity, which may enhance the release or conversion of HTyr-derived phenolic compounds, contributing to the elevated recovery values.

As for the emulsions, the bioaccessibility of HTyr displayed a variable pattern with a general increase throughout the digestive process. In this regard, the highest percentages of bioaccessibility (above 80%) were observed in the later gastric phases (60 and 120 min). While the oil matrix showed a higher initial content of HTyr compared to the emulsion, this may result in less efficient absorption due to the potential degradation of a substantial amount of the compound before it reaches the optimal absorption sites in the small intestine. In contrast, the emulsion matrix demonstrated a more gradual and sustained release of HTyr, with bioaccessibility steadily increasing throughout digestion. A controlled release, particularly evident in the late gastric phase, suggests the greater stability of HTyr in emulsions when exposed to the changing pH and enzymatic conditions during digestion, especially during the gastric–intestinal transition. This enhanced stability compared to EVOO is crucial for ensuring that a higher quantity of HTyr remains intact and reaches the intestinal phase, where absorption is typically more efficient. Thus, although the oil matrix may exhibit a higher initial recovery, the controlled and sustained bioaccessibility of HTyr in emulsions is likely to improve its overall bioavailability, making emulsions a more effective delivery system for maximizing HTyr’s beneficial effects. Furthermore, HTyr could reach the colon and be transformed into its metabolites by the colonic microbiota, exerting beneficial effects both locally and systemically [67,68,69]. Nevertheless, future studies are required to explore HTyr-controlled colonic release strategies to enhance its bioavailability and optimize its health-promoting effects in the lower gastrointestinal tract.

Concerning the phenolic extract, HTyr exhibited variable bioaccessibility throughout the digestive process. During the gastric phase, HTyr bioaccessibility remained relatively stable, with the highest value recorded after 30 min of gastric digestion (42.7%) and no significant differences until 120 min (*p* > 0.05). Maximum bioaccessibility was achieved at 180 min, reaching 52.4%, indicating efficient release and potential absorption during the early intestinal phase. However, a marked decline was observed at 240 min (28.4%), suggesting a significant reduction in bioaccessibility during the later stages of the intestinal phase, likely due to degradation or reduced solubility under prolonged intestinal conditions. Nevertheless, it was observed that throughout the entire digestive process, the percentage recovery of this matrix remained below 52%, which is lower than the values obtained for both the oil and the emulsion. This suggests that the food matrix itself and the encapsulation of HTyr in water droplets have protective effects on its stability and bioaccessibility. These results differ from those obtained by Duque-Soto et al. [70], where not only were higher recovery rates observed compared to those obtained in the present study, but there was also a dramatic increase in the recovery rate at the beginning of the intestinal phase. These differences could be attributed to variations in the experimental conditions, particularly in the extraction process and the composition of the extract used. Thus, the extraction methods, including the solvents and techniques applied, likely resulted in a different composition and stability of phenolic compounds, which may explain the higher recovery rates. Moreover, the composition of the extract employed in the previous research differed from that in the present study, potentially contributing to the pronounced increase in recovery during the initial intestinal phase.

Regarding OLE, the results obtained for the emulsion showed a significant increase in bioaccessibility during the early stages of the digestive process, particularly between 30 and 60 min of the gastric phase. However, the absence of significant differences between 60 and 120 min implies that it may reach a maximum or stabilize after 60 min. Moreover, in the stomach, the acidic environment cleaves the β-glycosidic bond of OLE, originating its aglycone form, which is transformed into two dialdehydes that are unstable and rapidly converted into a transposed secoiridoid, a lipophilic compound that under specific conditions, such as prolonged exposure to an acidic environment, can release a HTyr moiety [9]. Bioaccessibility values in the intestinal phase were generally lower than those observed in the gastric phase, however a trend towards stabilization was observed after 210 min.

In addition, the bioaccessibility profile of OLE in the phenolic extract followed a distinct pattern. During the gastric phase, bioaccessibility remained constant, ranging from 35.2% to 35.6%. Upon transitioning to the intestinal phase, a significant increase was observed, reaching values of up to 45.5% at 180 and 210 min. This indicates that the intestinal environment, particularly the enzymatic activity and solubilization processes, could enhance the passage of OLE from the phenolic extract to the bioaccessible fraction. However, bioaccessibility sharply decreased to 23.3% at 240 min, likely due to metabolic transformations during the later stages of digestion, resulting in reduced availability by the end of the intestinal phase. In fact, OLE is known to be highly sensitive to digestive degradation, with a different bioaccessibility profile compared to that observed in the emulsion, with values consistently below 50%, similar to the pattern observed for HTyr. These findings are consistent with those reported in a previous study in which a decrease in OLE bioaccessibility during the intestinal phase, along with similar percentage recovery values, was also observed [34].

## 4. Conclusions

As can be shown, the findings of this study highlight the potential of emulsification as an effective strategy for enhancing the stability, bioaccessibility, and overall retention of the phenolic compounds HTyr and OLE during digestion and storage. The incorporation of phenolic-rich extracts into EVOO using a W/O emulsion resulted in improved EE, particularly for OLE, and a more controlled, sustained release of HTyr throughout the digestive process compared to the phenolic extract or EVOO matrices. This gradual release of HTyr in the emulsion enhances its bioaccessibility, ensuring greater stability and protection against degradation, especially during the gastric–intestinal transition, which is crucial for maximizing its absorption in the small intestine. Additionally, the emulsion matrix proved to be superior in preserving secoiridoid compounds during storage, further supporting the idea that emulsification can enhance the functional properties of fortified formulations. Overall, these results demonstrate that emulsification is a promising green process for developing phenolic-rich products, optimizing the stability and bioaccessibility of key bioactive compounds for potential health benefits.

## Figures and Tables

**Table 1:** Compounds tentatively identified in the oil, extract, and emulsion samples.

Peak Number	Proposed Compound	Molecular Formula	RT ^1^ (min)	*m*/*z* Expe ^2^	*m*/*z* Theor ^3^	Error (ppm)	mSigma	Samples
1	Oxidized HTyr	C_8_H_8_O_3_	5.13	151.0403	151.0400	−2.2	16.1	Oil *, emulsion
2	HTyr glucoside	C_14_H_20_O_8_	8.50	315.1085	315.1085	0.2	27.3	Emulsion * and extract
3	HTyr	C_8_H_10_O_3_	9.12	153.0550	153.0557	−1.1	8	Oil *, emulsion, and extract
4	DEA	C_9_H_12_O_4_	10.08	183.0659	183.0663	2.3	7.4	Oil *, emulsion
5	Elenolic acid isomer 1	C_11_H_14_O_6_	10.93	241.0718	241.0717	−0.2	22.1	Oil *, emulsion, and extract
6	Tyrosol	C_8_H_10_O_2_	11.23	137.0603	136.0608	3.1	9.9	Oil *, emulsion, and extract
7	Oleoside/Secologanoside	C_16_H_22_O_11_	12.20	389.1079	389.1089	2.7	26	Emulsion * and extract
8	Glucosylated form of elenolic acid isomer 1	C_17_H_24_O_11_	12.54	403.1235	403.1246	2.6	14.6	Extract *
9	Hydroxy D-OLE aglycone	C_17_H_20_O_7_	13.11	335.1128	335.1136	2.2	33	Oil *, emulsion
10	Hydroxy OLE	C_25_H_32_O_14_	15.12	555.1710	555.1719	1.7	20.7	Emulsion * and extract
11	Demethyl OLE	C_24_H_30_O_13_	15.39	525.1594	525.1614	3.7	13.5	Extract *
12	Methyl oleoside/Methyl secologanoside	C_17_H_24_O_11_	15.75	403.1245	403.1246	0.1	13.1	Emulsion * and extract
13	Glucosylated form of elenolic acid isomer 2	C_17_H_24_O_11_	15.86	403.1243	403.1246	0.7	18.2	Emulsion * and extract
14	Hydrated product of OH-DOA	C_17_H_22_O_7_	16.00	337.1276	337.1293	4.9	41.7	Oil *, emulsion, and extract
15	HTyr acetate	C_10_H_12_O_4_	16.64	195.0666	195.0662	−2.1	13.1	Oil *, emulsion, and extract
16	Geminal diol OLE aglycone isomer 1	C_19_H_24_O_9_	16.85	395.1331	395.1348	4.1	7.8	Emulsion * and extract
17	Geminal diol OLE aglycone isomer 2	C_19_H_24_O_9_	17.10	395.1330	395.1348	4.5	10.4	Emulsion * and extract
18	Geminal diol OLE aglycone isomer 3	C_19_H_24_O_9_	17.56	395.1334	395.1348	3.5	17.9	Oil, emulsion *, and extract
19	Elenolic acid isomer 2	C_11_H_14_O_6_	18.00	241.0718	241.0717	−0.5	10.4	Oil *, emulsion, and extract
20	Luteolin 7-O-glucoside	C_21_H_20_O_11_	18.15	447.0895	447.0933	8.4	17	Emulsion * and extract
21	Hydroxyelenolic acid	C_11_H_14_O_7_	19.20	257.0670	257.0666	−1.3	13.4	Oil *, emulsion
22	DOA	C_17_H_20_O_6_	19.58	319.1182	319.1187	1.5	24.3	Oil *, emulsion, and extract
23	OLE aglycone isomer 1	C_19_H_22_O_8_	19.60	377.1243	377.1241	−0.3	9.9	Oil *, emulsion
24	OLE aglycone isomer 2	C_19_H_22_O_8_	20.00	377.1242	377.1241	−0.1	6.4	Oil *, emulsion
25	OLE aglycone isomer 3	C_19_H_22_O_8_	20.40	377.1233	377.1241	2.1	12.9	Oil *, emulsion
26	OLE aglycone isomer 4	C_19_H_22_O_8_	20.80	377.1246	377.1241	−1.2	3.9	Oil *, emulsion
27	OLE	C_25_H_32_O_13_	21.10	539.1715	539.1711	−0.7	44.6	Emulsion * and extract
28	Chrysoeriol 7-O-glucoside/Diosmetin 7-O-glucoside	C_22_H_22_O_11_	21.42	461.1095	461.1089	−1.2	8.2	Emulsion * and extract
29	Syringaresinol	C_22_H_26_O_8_	21.74	417.1555	417.1554	−0.2	22.7	Oil *, emulsion
30	10-hydroxy OLE aglycone	C_19_H_22_O_9_	22.50	373.1197	373.1191	−1.7	24.1	Oil *, emulsion
31	Pinoresinol	C_20_H_22_O_6_	22.76	357.1343	357.1343	−0.1	31	Oil *, emulsion
32	OLE isomer 1	C_25_H_32_O_13_	23.40	539.1718	539.1711	−1.2	14.2	Emulsion * and extract
33	Acetoxypinoresinol	C_22_H_24_O_8_	23.45	415.1382	415.1398	3.9	27.5	Oil *, emulsion
34	Ligstroside aglycone isomer 1	C_19_H_22_O_7_	23.50	361.1286	361.1292	1.6	1.9	Oil *, emulsion
35	Decarboxymethyl ligstroside aglycone	C_17_H_20_O_5_	23.54	303.1235	303.1237	0.9	10.6	Oil *, emulsion
36	Ligstroside aglycone isomer 2	C_19_H_22_O_7_	23.80	361.1286	361.1292	1.7	8.2	Oil *, emulsion
37	Ligstroside	C_25_H_32_O_12_	24.16	523.1363	523.1762	−0.1	60.5	Emulsion * and extract
38	Ligstroside aglycone isomer 3	C_19_H_22_O_7_	24.20	361.1287	361.1292	1.5	10.4	Oil *, emulsion
39	Methyl D-OLE aglycone	C_18_H_22_O_6_	24.50	333.1348	333.343	−1.6	1.1	Oil *, emulsion
40	Ligstroside aglycone isomer 4	C_19_H_22_O_7_	24.50	361.1292	361.1292	0.1	6.7	Oil *, emulsion
41	OLE isomer 2	C_25_H_32_O_13_	24.60	539.1729	539.1711	−3.2	45.4	Emulsion * and extract
42	OLE aglycone isomer 5	C_19_H_22_O_8_	25.30	377.1240	377.1241	0.3	20.4	Oil *, emulsion
43	Dehydro OLE aglycone	C_19_H_20_O_8_	25.30	375.1081	375.1085	1.0	22.1	Oil *, emulsion
44	OLE aglycone isomer 6	C_19_H_22_O_8_	26.40	377.1242	377.1241	0	20	Oil *, emulsion
45	Luteolin	C_15_H_10_O_6_	26.75	285.0413	285.0404	−3.1	8.7	Oil *, emulsion
46	OLE aglycone isomer 7	C_19_H_22_O_8_	27.10	377.1240	377.1241	0.4	20.3	Oil *, emulsion
47	Ligstroside aglycone isomer 5	C_19_H_22_O_7_	27.70	361.1294	361.1292	−0.5	3	Oil *, emulsion
48	Apigenin	C_15_H_10_O_5_	28.10	269.0453	269.0455	0.6	12.2	Oil *, emulsion
49	Ligstroside aglycone isomer 6	C_19_H_22_O_7_	28.10	361.1297	361.1292	−1.4	16.5	Oil *, emulsion
50	Ligstroside aglycone isomer 7	C_19_H_22_O_7_	28.30	361.1293	361.1292	−0.1	6.4	Oil *, emulsion

* Sample used to obtain the values of experimental and theoretical *m*/*z*, error, and mSigma; ^1^: Retention time; ^2^: Experimental; ^3^: Theoretical; DOA: Decarboxymethyl oleuropein aglycone; DEA: Decarboxymethylated elenolic acid; Hydroxytyrosol: HTyr; Oleuropein: OLE.

**Table 2:** Quantification of compounds identified in olive oil, leaf extract, and W/O emulsion.

	Olive Oils (mg Compound/kg Oil)	Emulsions (mg Compound/kg Emulsion)	Leaf Extract (mg Compound/kg Extract)
**Total phenolic alcohols**	**24 ± 2 ^a^**	**239 ± 10 ^b^**	**244 ± 11**
HTyr	14 ± 2 ^a^	220 ± 14 ^b^	223 ± 10
Oxidized HTyr	0.396 ± 0.004 ^a^	0.801 ± 0.001 ^b^	ND
HTyr glucoside	ND	4.50 ± 0.15	17.80 ± 0.85
HTyr acetate	1.4 ± 0.3 ^a^	3.6 ± 0.3 ^b^	1.205 ± 0.005
Tyrosol	7.7 ± 0.7 ^a^	9.3 ± 0.1 ^b^	2.005 ± 0.005
**Total secoiridoids**	**590 ± 21 ^a^**	**643 ± 12 ^b^**	**493 ± 17**
OLE	ND	89.6 ± 0.5	422 ± 13
OLE isomer 1	ND	1.602 ± 0.002	11.0 ± 0.7
OLE isomer 2	ND	6.3 ± 0.1	34 ± 1
Hydroxy OLE	ND	2 × 10^−2^ ± 2 × 10^−5^	1.0 ± 0.1
Demethyl OLE	ND	NC	0.52 ± 0.06
Hydroxy D-OLE aglycone	0.7 ± 0.1 ^a^	0.36 ± 0.03 ^b^	ND
10-hydroxy OLE aglycone	3 ± 1 ^a^	2.9 ± 0.1 ^a^	ND
Geminal diol OLE aglycone 1	NQ	3.5 ± 0.5	3.4 ± 0.2
Geminal diol OLE aglycone 2	NQ	4.9 ± 0.5	4.8 ± 0.2
Geminal diol OLE aglycone 3	0.257 ± 0.002 ^a^	6.4 ± 0.4 ^b^	6.9 ± 0.5
Dehydro OLE aglycone	4.5 ± 0.2 ^a^	4.2 ± 0.3 ^a^	ND
Methyl D-OLE aglycone	27.8 ± 5.6 ^a^	20.49 ± 1.09 ^b^	ND
DOA	72 ± 12 ^a^	69 ± 6 ^a^	0.721 ± 0.001
Hydrated product of OH-DOA	0.9 ± 0.2 ^a^	3.8 ± 0.4 ^b^	2.1 ± 0.1
Ligstroside	ND	1.93 ± 0.01	5.5 ± 0.2
Decarboxymethyl ligstroside aglycone	29 ± 9 ^a^	26 ± 3 ^a^	ND
OLE aglycone isomer	386 ± 2 ^a^	359 ± ^b^ 2	ND
Ligstroside aglycone isomer	55 ± 11 ^a^	24 ± 2 ^b^	ND
**Total lignans**	**11.9 ± 0.3 ^a^**	**11.2 ± 0.2 ^b^**	**ND**
Pinoresinol	7.0 ± 0.3 ^a^	6.5 ± 0.1 ^b^	ND
Acetoxypinoresinol	3.96 ± 0.05 ^a^	3.81 ± 0.01 ^b^	ND
Syringaresinol	0.9 ± 0.1 ^a^	0.9 ± 0.1 ^a^	ND
**Total flavonoids**	**10.4 ± 0.4 ^a^**	**13 ± 1 ^b^**	**10 ± 0.3**
Apigenin	2.18 ± 0.03 ^a^	2.2 ± 0.3 ^a^	NQ
Luteolin	8.2 ± 0.6 ^a^	8.2 ± 0.6 ^a^	NQ
Luteolin 7-O-glucoside	ND	2.402 ± 0.002	9.8 ± 0.3
**Total oleosides and elenolic acid derivates**	**58.0 ± 0.3 ^a^**	**96 ± 9 ^b^**	**62 ± 3**
Elenolic acid isomer 1	11 ± 1 ^a^	11.9 ± 0.9 ^a^	3.0 ± 0.1
Elenolic acid isomer 2	33 ± 14 ^a^	77 ± 11 ^b^	9.6 ± 0.4
Hydroxyelenolic acid	1.7 ± 0.4 ^a^	0.9 ± 0.1 ^b^	ND
DEA	0.3 ± 0.1 ^a^	4 × 10^−1^ ± 4 × 10^−4 a^	0.22 ± 0.03
Glucosylated form of elenolic acid isomer 1	ND	NQ	0.60 ± 0.06
Glucosylated form of elenolic acid isomer 2	ND	2.1 ± 0.1	10.2 ± 0.4
Oleoside/Secologanoside	ND	1.44 ± 0.03	11.6 ± 0.5
Methyl oleoside/Methyl secologanoside	ND	2.10 ± 0.06	9.9 ± 0.4
**Total phenolic compounds**	**637 ± 24 ^a^**	**907 ± 23 ^b^**	**747 ± 28**

ND = not detected; NQ = not quantified; values are the means ± SD (n = 3); different letters indicate a statistically significant difference between the two matrices (*p* < 0.05); DOA: Decarboxymethyl oleuropein aglycone; DEA: Decarboxymethylated elenolic acid; Hydroxytyrosol: HTyr; Oleuropein: OLE.

## Data Availability

All the data generated by this research have been included in the article. For any assistance, it is possible to contact the corresponding authors.

## Supplementary Materials

The following supporting information can be downloaded at https://www.mdpi.com/article/10.3390/nu16223909/s1: Table S1: Standards and calibration curves used in the quantification of olive oil compounds. Table S2: Quantification of compounds identified in olive oil and W/O emulsion over time. Concentrations are expressed as mean ± standard deviation of three replicates in mg/kg.
