# Supplementary material for: Functional Olive Oil Production via Emulsions: Evaluation of Phenolic Encapsulation Efficiency, Storage Stability, and Bioavailability
Source: Nutrients. 2024 Nov 15;16(22):3909. doi: 10.3390/nu16223909 (PMC11597669; doi:10.3390/nu16223909)
Supplement: Supplementary file 1 [file nutrients-16-03909-s001.zip › nutrients-3304153-supplementary.pdf]

Table S1. Standards and calibration curves used in the quantification of phenolic compounds.

Standard	Calibration Curve	R^2
Hydroxytyrosol	y = 36726x + 124763	0.992
Oleuropein	y = 102573x + 7821	0.982
Tyrosol	y = 20027x + 511	0.996
Pinoresinol	y = 27862x + 57	0.996
Luteolin-7-glucoside	y = 73590x + 2525	0.991
Apigenin	y = 164399x + 143280	0.997

Table S2. Quantification of compounds identified in olive oil and W/O emulsion over time. Concentrations are expressed as mean ± standard deviation of three replicates in mg/kg.

	**EVOO**						**W/O Emulsion**					
**Days of storage**	**0**	**4**	**8**	**12**	**16**	**19**	**0**	**4**	**8**	**12**	**16**	**19**
**Total phenolic alcohols**	**24 ± 2** * ^{a}	**25 ± 2** * ^{a}	**21 ± 1** * ^{a}	**24.3 ± 0.1** * ^{a}	**24.5 ± 0.9** * ^{a}	**22 ± 1** * ^{a}	**239 ± 10** # ^{a}	**210 ± 4** # ^{bc}	**226 ± 12** # ^{ab}	**215.8 ± 0.3** # ^{bc}	**213 ± 3** # ^{bc}	**202 ± 6** # ^{c}
Hydroxytyrosol	14 ± 2 * ^{a}	15 ± 2 * ^{a}	12 ± 1 * ^{a}	14.37 ± 0.01 * ^{a}	15 ± 1 * ^{a}	13 ± 1 * ^{a}	221 ± 10 # ^{a}	192 ± 4 # ^{bc}	207 ± 10 # ^{ab}	197 ± 1 # ^{bc}	192 ± 2 # ^{bc}	182 ± 6 # ^{c}
Oxidized hydroxytyrosol	0.396 ± 0.004 * ^{a}	0.5 ± 0.1 * ^{a}	$4x10^{-1}$ ± $6x10^{-5}$ * ^{a}	0.40 ± 0.01 * ^{a}	0.396 ± 0.001 * ^{a}	$4x10^{-1}$ ± $4x10^{-4}$ * ^{a}	0.801 ± 0.001 # ^{b}	0.798 ± 0.001 # ^{b}	1.1 ± 0.1 # ^{ab}	0.9 ± 0.1 # ^{bc}	1.0 ± 0.1 # ^{ab}	1.198 ± 0.001 # ^{a}
Hydroxytyrosol glucoside	ND	ND	ND	ND	ND	ND	4.5 ± 0.1 ^{c}	5.9 ± 0.3 ^{bc}	4.9 ± 0.7 ^{c}	5 ± 1 ^{bc}	7.55 ± 0.01 ^{a}	6.5 ± 0.1 ^{a}
Hydroxytyrosol acetate	1.4 ± 0.2 * ^{b}	1.6 ± 0.2 * ^{a}	1.3 ± 0.1 * ^{ab}	1.394 ± 0.006 * ^{ab}	1.39 ± 0.01 * ^{ab}	1.1 ± 0.1 * ^{a}	3.6 ± 0.2 # ^{a}	2.9 ± 0.1 # ^{bc}	3.1 ± 0.3 # ^{b}	3.1 ± 0.1 # ^{b}	2.8 ± 0.2 # ^{bc}	2.5 ± 0.1 # ^{c}
Tyrosol	8 ± 1 * ^{a}	8 ± 1 * ^{a}	7.2 ± 0.4 * ^{a}	8.1 ± 0.1 * ^{a}	7.9 ± 0.2 * ^{a}	7.2 ± 0.2 * ^{a}	9.3 ± 0.1 # ^{c}	8.8 ± 0.2 * ^{c}	10.0 ± 0.6 # ^{ab}	10.2 ± 0.2 # ^{ab}	10.5 ± 0.6 # ^{a}	10.4 ± 0.3 # ^{a}
**Tota secoiridoids**	**590 ± 21** * ^{a}	**603 ± 27** * ^{a}	**580 ± 10** * ^{a}	**588 ± 3** * ^{a}	**583 ± 22** * ^{a}	**444 ± 20** * ^{b}	**643 ± 12** # ^{a}	**619 ± 6** * ^{b}	**612 ± 5** # ^{b}	**604 ± 3** # ^{b}	**564 ± 6** * ^{c}	**572 ± 3** # ^{c}
Oleuropein	ND	ND	ND	ND	ND	ND	89.6 ± 0.4 ^{c}	96 ± 1 ^{bc}	92.3 ± 0.4 ^{bc}	98 ± 6 ^{ab}	95 ± 1 ^{bc}	103 ± 2 ^{a}
Oleuropein isomer 1	ND	ND	ND	ND	ND	ND	1.6002 ± 0.002 ^{ab}	1.7 ± 0.1 ^{a}	1.199 ± 0.004 ^{c}	1.3 ± 0.3 ^{bc}	1.34 ± 0.05 ^{abc}	1.39 ± 0.01 ^{abc}
Oleuropein isomer 2	ND	ND	ND	ND	ND	ND	6.3 ± 0.1^{ab}	6.6 ± 0.2 ^{a}	5.4 ± 0.2 ^{b}	5 ± 1 ^{b}	5.6 ± 0.2 ^{b}	5.9 ± 0.1^{ab}
Hydroxy oleuropein	ND	ND	ND	ND	ND	ND	$2x10^{-2}$ ± $2x10^{-5}$ ^{d}	0.16 ± 0.04 ^{bc}	0.01 ± 0.01 ^{d}	0.07 ± 0.07 ^{cd}	0.29 ± 0.03 ^{a}	0.19 ± 0.01 ^{b}
Demethyl oleuropein	ND	ND	ND	ND	ND	ND	NQ	NQ	NQ	NQ	NQ	NQ

Hydroxy D-oleuropein aglycone	0.7 ± 0.1 * [ab]	0.8 ± 0.1 * [a]	0.74 ± 0.04 * [b]	0.75 ± 0.01 * [ab]	0.71 ± 0.04 * [ab]	0.58 ± 0.04 * [b]	0.36 ± 0.02 # [a]	0.23 ± 0.01 # [b]	0.25 ± 0.03 # [b]	0.13 ± 0.03 # [c]	0.20 ± 0.01 # [b]	0.13 ± 0.01 # [c]
10-hydroxy oleuropein aglycone	3.0 ± 0.8 * [a]	3.2 ± 0.4 * [a]	3 ± 1 * [a]	2.595 ± 0.001 * [a]	3.4 ± 0.2 * [a]	2.6 ± 0.2 * [a]	2.9 ± 0.1 * [bc]	2.991 ± 0.003 * [c]	3.4 ± 0.2 * [a]	2.6 ± 0.2 * [cd]	2.58 ± 0.01 # [cd]	2.5 ± 0.1 # [c]
Geminal diol oleuropein aglycone 1	NQ	NQ	NQ	NQ	NQ	NQ	3.5 ± 0.3 [a]	2.67 ± 0.02 [b]	2.7 ± 0.2 [b]	1.57 ± 0.04 [c]	1.57 ± 0.04 [c]	1.24 ± 0.09 [c]
Geminal diol oleuropein aglycone 2	NQ	NQ	NQ	NQ	NQ	NQ	5.0 ± 0.4 [a]	3.38 ± 0.01 [b]	2.9 ± 0.1 [c]	1.7 ± 0.1 [d]	1.5 ± 0.1 [d]	0.90 ± 0.05 [e]
Geminal diol oleuropein aglycone 3	0.257 ± 0.002 * [ab]	0.38 ± 0.08 * [a]	0.21 ± 0.05 * [b]	0.31 ± 0.01 * [ab]	0.28 ± 0.04 * [ab]	0.24 ± 0.06 * [b]	6.4 ± 0.3 # [a]	4.76 ± 0.07 # [b]	4.6 ± 0.1 # [b]	2.98 ± 0.05 # [c]	2.87 ± 0.05 # [c]	2.2 ± 0.1 # [d]
Dehydro oleuropein aglycone	4.5 ± 0.2 * [ab]	4.9 ± 0.1 * [a]	4.4 ± 0.2 * [b]	4.79 ± 0.01 [a] [ab]	4.7 ± 0.1 * [ab]	4.6 ± 0.2 *[ab]	4.2 ± 0.2 * [abc]	4.187 ± 0.004 # [bc]	4.5 ± 0.1 * [a]	4.5 ± 0.1 # [ab]	4.3 ± 0.1 # [abc]	4.171 ± 0.004 # [c]
Methyl D-oleuropein aglycone	28 ± 4 * [a]	28 ± 3 * [a]	22 ± 1 * [a]	24.0 ± 0.1 * [a]	25 ± 1 * [a]	22 ± 1 * [a]	20 ± 1 # [a]	19.4 ± 0.1 # [ab]	20 ± 1 # [ab]	18.9 ± 0.3 # [b]	16.2 ± 0.5 # [c]	16 ± 1 # [c]
DOA	72 ± 8 * [abc]	82 ± 7 * [a]	72 ± 5 * [bc]	73.38 ± 0.07 * [abc]	69 ± 5 * [c]	56 ± 5 * [a]	70 ± 4 * [a]	30 ± 1 # [c]	41 ± 4 # [b]	26.9 ± 0.9 # [c]	26 ± 3 # [c]	22.5 ± 0.4 # [c]
Hydrated product of OH-DOA	1.0 ± 0.1 * [ab]	1.1 ± 0.2 * [a]	0.9 ± 0.1 * [b]	0.97 ± 0.01 * [ab]	0.9 ± 0.1 * [ab]	0.78 ± 0.06 * [b]	3.8 ± 0.3 # [a]	3.03 ± 0.02 # [bc]	3.3 ± 0.2 # [b]	2.56 ± 0.03 # [de]	2.725 ± 0.002 # [cd]	2.1 ± 0.2 # [e]
Ligstroside	ND	ND	ND	ND	ND	ND	1.93 ± 0.01 [a]	1.73 ± 0.04 [bc]	1.46 ± 0.02 [cd]	1.3 ± 0.2 [c]	1.7 ± 0.1 [cb]	1.50 ± 0.07 [abc]
Decarboxymethyl ligstroside aglycone	29 ± 6 * [ab]	35 ± 5 * [a]	23 ± 1 * [b]	30.1 ± 0.3 * [ab]	26 ± 3 * [ab]	23 ± 1 * [b]	26 ± 2 * [a]	13.9 ± 0.2 # [b]	16 ± 1 # [b]	15.0 ± 0.2 # [b]	14.5 ± 0.3 # [b]	13.654 ± 0.004 # [b]
Oleuropein aglycone isomer	386 ± 2 * [a]	381 ± 9 * [a]	388.2 ± 0.3 * [a]	389 ± 1 * [a]	392 ± 7 * [a]	292 ± 14 * [b]	359 ± 2 # [d]	385 ± 9 # [a]	372.8 ± 0.1 # [bc]	379 ± 3 # [ab]	360 ± 1 # [d]	363 ± 4 # [d]

Ligstroside aglycone isomer	65 ± 1 * ^{a}	67 ± 3 * ^{a}	66 ± 3 * ^{a}	62 ± 2 * ^{a}	62 ± 4 * ^{a}	42.3 ± 0.1 # ^{b}	42 ± 1 # ^{ab}	44.0 ± 0.2 # ^{a}	41 ± 1 # ^{b}	42.2 ± 0.2 # ^{ab}	27.9 ± 0.4 # ^{d}	31.9 ± 0.6 # ^{c}
**Total lignans**	**11.9 ± 0.3** * ^{abc}	**12.6 ± 0.4** * ^{a}	**11.7 ± 0.3** * ^{ab}	**12.3 ± 0.3** * ^{bc}	**11.7 ± 0.4** * ^{ab}	**11.41 ± 0.01** * ^{a}	**11.2 ± 0.2** # ^{c}	**12.1 ± 0.1** # ^{b}	**12.6 ± 0.2** # ^{a}	**12.4 ± 0.2** * ^{ab}	**11.6 ± 0.1** * ^{c}	**11.4 ± 0.1** * ^{c}
Pinoresinol	7.0 ± 0.2 * ^{abc}	7.5 ± 0.3 * ^{a}	6.8 ± 0.2 * ^{bc}	7.3 ± 0.1 * ^{ab}	6.8 ± 0.3 * ^{bc}	6.5 ± 0.1 * ^{c}	6.5 ± 0.1 * ^{d}	7.18 ± 0.01 # ^{bc}	7.6 ± 0.2 # ^{a}	7.4 ± 0.2 * ^{ab}	6.9 ± 0.1 * ^{d}	6.7 ± 0.1 * ^{d}
Acetoxypinoresinol	3.96 ± 0.04 * ^{b}	4.196 ± 0.004 * ^{a}	4.005 ± 0.005 * ^{b}	4.1 ± 0.1 * ^{ab}	3.96 ± 0.01 * ^{b}	4.1 ± 0.1 * ^{ab}	3.81 ± 0.01 # ^{c}	4.187 ± 0.004 * ^{ab}	4.20 ± 0.01 # ^{a}	4.18 ± 0.01 * ^{ab}	4.17 ± 0.01 * ^{b}	4.17 ± 0.01 * ^{b}
Syringaresinol	0.9 ± 0.1 * ^{a}	0.9 ± 0.1 * ^{a}	0.9 ± 0.1 * ^{a}	0.9 ± 0.1 * ^{a}	0.9 ± 0.1 * ^{a}	0.801 ± 0.001 * ^{a}	0.9 ± 0.1 * ^{a}	0.7 ± 0.1 ^{bc}	0.799 ± 0.002 * ^{ab}	0.79 ± 0.01 * ^{ab}	0.59 ± 0.01 # ^{c}	0.59 ± 0.01 # ^{c}
**Total flavonoids**	**10.4 ± 0.4** * ^{ab}	**11.9 ± 0.3** * ^{a}	**10 ± 1** * ^{b}	**11.0 ± 0.6** * ^{ab}	**11.1 ± 0.7** * ^{ab}	**10 ± 1** * ^{b}	**13 ± 1** # ^{b}	**12.9 ± 0.3** # ^{b}	**13 ± 1** * ^{b}	**12.5 ± 0.1** * ^{ab}	**13 ± 1** * ^{ab}	**10 ± 1** * ^{a}
Apigenin	2.18 ± 0.02 * ^{a}	2.398 ± 0.002 * ^{a}	2.1 ± 0.3 * ^{a}	2.2 ± 0.2 * ^{a}	2.2 ± 0.4 * ^{a}	2.1 ± 0.1 * ^{a}	2.2 ± 0.2 * ^{a}	2.1 ± 0.1 # ^{a}	2.1 ± 0.1 * ^{a}	2.1 ± 0.1 * ^{a}	1.9 ± 0.3 * ^{ab}	1.6 ± 0.2 * ^{b}
Luteolin	8.2 ± 0.4 * ^{ab}	9.5 ± 0.3 * ^{a}	8 ± 1 * ^{b}	8.8 ± 0.4 * ^{ab}	9 ± 1 * ^{ab}	7.5 ± 0.5 * ^{b}	8.2 ± 0.4 * ^{a}	7.9 ± 0.1 # ^{a}	8.5 ± 0.5 * ^{a}	8.0 ± 0.4 * ^{a}	7 ± 1 * ^{ab}	6.3 ± 0.7 * ^{b}
Luteolin-7-glucoside	ND	ND	ND	ND	ND	ND	2.402 ± 0.002 ^{b}	2.9 ± 0.1 ^{ab}	2.5 ± 0.3 ^{b}	2.4 ± 0.4 ^{b}	3.5 ± 0.3 ^{a}	2.582 ± 0.003 ^{b}
**Total oleosides and elenolic acid derivates**	**58.0 ± 0.3** * ^{a}	**50 ± 3** * ^{b}	**36 ± 2** * ^{d}	**43.5 ± 0.8** * ^{b}	**46 ± 3** * $^{b, c}$	**45 ± 1** * ^{b}	**96 ± 9** # ^{d}	**114 ± 6** # ^{c}	**147 ± 3** # ^{b}	**161 ± 2** # ^{a}	**157 ± 4** # ^{ab}	**146 ± 1** # ^{b}
Elenolic acid isomer 1	11 ± 1 * ^{a}	12 ± 1 * ^{a}	11.1 ± 0.4 * ^{a}	11.0 ± 0.1 * ^{a}	12 ± 1 * ^{a}	12.1 ± 0.4 * ^{a}	12 ± 1 * ^{a}	12.0 ± 0.2 * ^{a}	13 ± 1 # ^{a}	11.4 ± 0.3 # ^{ab}	11.7 ± 0.3 * ^{ab}	10.3 ± 0.6 * ^{b}
Elenolic acid isomer 2	45 ± 1 * ^{a}	36 ± 1 * ^{b}	23 ± 2 * ^{d}	31 ± 1 * ^{b}	32 ± 3 * ^{bc}	31.1 ± 0.3 * ^{c}	77 ± 8 # ^{d}	91 ± 5 # ^{c}	124.25 ± 0.01 # ^{b}	139 ± 4 # ^{a}	130 ± 5 # ^{ab}	122.56 ± 0.01 # ^{a}
Hydroxyelenolic acid	1.7 ± 0.3 * ^{a}	1.8 ± 0.2 * ^{a}	1.4 ± 0.2 * ^{a}	1.796 ± 0.001 * ^{a}	1.4 ± 0.2 * ^{a}	1.3 ± 0.1 * ^{a}	0.9 ± 0.1 # ^{d}	1.196 ± 0.001 # ^{cd}	1.6 ± 0.2 * ^{b}	1.5 ± 0.1 # ^{bc}	2.4 ± 0.2 # ^{a}	2.1 ± 0.1 # ^{a}
DEA	0.3 ± 0.1 * ^{a}	0.3 ± 0.1 * ^{a}	$2x10^{-1}$ ± $6x10^{-5}$ * ^{a}	0.199 ± 0.001 * ^{a}	0.198 ± 0.001 * ^{a}	$2x10^{-1}$ ± $2x10^{-4}$ * ^{a}	$4x10^{-1}$ ± $4x10^{-4}$ * ^{a}	$4x10^{-1}$ ± $4x10^{-4}$* ^{a}	0.5 ± 0.1 # ^{a}	0.40 ± 0.01# ^{a}	0.40 ± 0.01 # ^{a}	0.20 ± 0.01 * ^{b}
Glucosylated form of elenolic acid isomer 1	ND	ND	ND	ND	ND	ND	NQ	0.1 ± 0.1 ^{c}	0.1 ± 0.1 ^{d}	0.1 ± 0.1 ^{cd}	0.399 ± 0.001 ^{a}	0.198 ± 0.001 ^{ab}

Glucosylated form of elenolic acid isomer 2	ND	ND	ND	ND	ND	ND	2.1 ± 0.1 [d]	3.2 ± 0.2 [bc]	2.8 ± 0.4 [cd]	2.9 ± 0.5 [cd]	4.2 ± 0.2 [a]	3.9 ± 0.1 [ab]
Oleoside/secologan oside	ND	ND	ND	ND	ND	ND	1.44 ± 0.02 [d]	2.9 ± 0.2 [abc]	2.3 ± 0.5 [cd]	2.5 ± 0.6 [bc]	3.7 ± 0.3 [a]	3.3 ± 0.1 [ab]
Methyl oleoside / Methyl secologanoside	ND	ND	ND	ND	ND	ND	2.10 ± 0.04 [d]	3.2 ± 0.1 [bc]	2.8 ± 0.4 [cd]	2.9 ± 0.5 [cd]	4.2 ± 0.3 [a]	3.8 ± 0.1 [ab]

ND= not detected, NQ= not quantified; EVOO= extra virgin olive oil. Different letters indicate significant differences between each time within each sample ($p<0.05$). Different symbols indicate a statistically significant difference between the two matrices on each day of storage ($p<0.05$). Decarboxymethyl oleuropein aglycone: DOA; Decarboxymethylated elenolic acid: DEA
